# Using Fiction to Assess Mental State Understanding: A New Task for Assessing Theory of Mind in Adults

**DOI:** 10.1371/journal.pone.0081279

**Published:** 2013-11-07

**Authors:** David Dodell-Feder, Sarah Hope Lincoln, Joseph P. Coulson, Christine I. Hooker

**Affiliations:** 1 Department of Psychology, Harvard University, Cambridge, Massachusetts, United States of America; 2 No Affiliation, Cambridge, Massachusetts, United States of America; Boston College, United States of America

## Abstract

Social functioning depends on the ability to attribute and reason about the mental states of others – an ability known as theory of mind (ToM). Research in this field is limited by the use of tasks in which ceiling effects are ubiquitous, rendering them insensitive to individual differences in ToM ability and instances of subtle ToM impairment. Here, we present data from a new ToM task – the Short Story Task (SST) - intended to improve upon many aspects of existing ToM measures. More specifically, the SST was designed to: (a) assess the full range of individual differences in ToM ability without suffering from ceiling effects; (b) incorporate a range of mental states of differing complexity, including epistemic states, affective states, and intentions to be inferred from a first- and second-order level; (c) use ToM stimuli representative of real-world social interactions; (d) require participants to utilize social context when making mental state inferences; (e) exhibit adequate psychometric properties; and (f) be quick and easy to administer and score. In the task, participants read a short story and were asked questions that assessed explicit mental state reasoning, spontaneous mental state inference, and comprehension of the non-mental aspects of the story. Responses were scored according to a rubric that assigned greater points for accurate mental state attributions that included multiple characters’ mental states. Results demonstrate that the SST is sensitive to variation in ToM ability, can be accurately scored by multiple raters, and exhibits concurrent validity with other social cognitive tasks. The results support the effectiveness of this new measure of ToM in the study of social cognition. The findings are also consistent with studies demonstrating significant relationships among narrative transportation, ToM, and the reading of fiction. Together, the data indicate that reading fiction may be an avenue for improving ToM ability.

## Introduction

 Navigation of the social world depends on one’s ability to make inferences about the mental life of others. Accurate understanding of another individual’s beliefs, emotions, intentions, and desires allows for the prediction of future mental states, associated actions, and engagement in appropriate social behavior. The importance of the mechanism that allows for mental state attribution, known as theory of mind (ToM), is perhaps best illustrated by cases in which ToM is impaired, as in schizophrenia and autism spectrum disorders [1-3]. In both of these disorders, ToM impairment carries functional and clinical significance in that the extent of ToM impairment is associated with the extent of dysfunction in social behavior [4-6]. Furthermore, in schizophrenia, improving ToM ability through targeted intervention is associated with improvements in aspects of real-world functioning [7-12]. In addition to its obvious clinical relevance, ToM underlies myriad social processes including compassion, sympathy, and empathy [13-15], moral judgment [16-21], negotiation [22], and marital/romantic relationship adjustment [23,24], among others. 

 One challenge confronting researchers studying ToM in adults is how to assess ToM accurately and reliably in a way that is sensitive to both subtle individual differences and clinical impairment. The most commonly used or “classic” ToM tasks [25], including the False-Belief Task [26-28], Hinting Task [29], Strange Stories Task [30], Faux Pas Task [31,32], Cartoon-Sequencing tasks [26,33,34], variations on the Heider and Simmel task [35-37], and the Reading the Mind in the Eyes Task (Eyes Task) [38] have been used successfully to distinguish clinical populations, such as individuals with schizophrenia [1-3], autism spectrum disorders [3], bipolar disorder [39-41], and individuals with brain damage to prefrontal cortex [31,42] and temporo-parietal junction [43,44], from healthy control participants. However, except in these aforementioned cases of severe ToM impairment, these tasks are insensitive to more subtle ToM deficits, let alone normal variation in ToM ability (although see below for a discussion of the Eyes Task, which does appear to be more sensitive to individual differences). Ceiling effects – in which participants perform at 100% or near 100% accuracy – are ubiquitously observed with these tasks, and their variants, in healthy control participants as well as patient groups (although less often; e.g., [29,31,32,34,36,39,45-59]). For example, in studies investigating ToM in schizophrenia, using papers identified in two meta-analyses [1,3], the comparison group of healthy control participants scored >90% accuracy in 6 of 7 studies using the Hinting Task [29,54,60-63] and 5 of 7 studies using the Faux Pas Task [64-68]. Clearly, these tasks are inadequate for addressing questions related to individual differences and normal variation in ToM ability. This inevitably limits the scope of questions researchers can ask about ToM ability and social behavior in adults. For example, ToM deficits have been observed in unaffected first-degree relatives of individuals with schizophrenia [58,69-72] and autism spectrum disorders [73-76], as well as individuals exhibiting attenuated symptoms of schizophrenia, but do not meet diagnostic criteria for a psychotic disorder [77-79]. Deficits in these “at-risk” groups have led researchers to propose ToM impairment as a vulnerability marker for these disorders [41,72], specifically that the presence of ToM deficits may reflect dysfunction in underlying neural circuitry associated with liability for the disorder. The negative consequences of ToM deficits, such as social conflict and social isolation, might also indirectly contribute to the development and onset of illness in populations at-risk. Tasks that do not adequately assess the full range of ToM abilities limit the potential to test ToM in these populations, in which deficits, when they do exist, are subtle, hard to detect, and yet may carry important implications regarding risk for psychopathology [72]. The ability to detect subtle impairment would bolster early identification and prevention efforts, and make ToM assessment a very useful clinical tool.

 There are several reasons as to why extant ToM measures lack sensitivity. For one, many of these tasks are adaptions of measures used to assess ToM skills in children [32,80-82]. As a consequence, the stimuli used may not be challenging enough for older individuals with more developed conceptual knowledge, reasoning skills, and social experience. Researchers increase the difficulty of ToM tasks by increasing the complexity of the mental state information, for example, by asking participants to make second-order (and higher) mental state inferences where mental states are embedded within other mental states (e.g., “Barbara thought that Hank knew where she thought her Yiddish dictionary was.”). This approach does indeed make tasks more challenging [83], but with greater complexity comes greater demands on non-social aspects of cognition including executive function, working memory, and verbal ability [84]. With these greater non-social demands, it becomes difficult to interpret performance as a function of ToM ability or non-social cognitive ability. Another important consideration is the context in which the participant is asked to make mental state inferences [25]. Are participants asked about the mental state of a single character that has a false-belief regarding the location of their chocolate bar? Or are participants asked questions about the mental state of characters involved in an ongoing dynamic social interaction embedded within a social context that requires the participant to apply their knowledge of social rules and contingencies? The latter is clearly more representative of mental state attributions made during real-world social interactions, and yet not at all representative of the stimuli used in “classic” ToM tasks. One final consideration is the distinction between implicit and spontaneous (i.e., considering mental state information without being prompted to do so) versus explicit and evoked mental state attributions [85]. Just about all of the standard ToM tasks ask participants to make explicit, reasoned mental state attributions that require considerable effort. Variations on the Heider and Simmel task, in which participants are asked to watch animated geometric figures move with or without ostensible intent and answer simply “What happened in the cartoon?” may be the exception [35,36]. The dissociation between implicit and explicit processes has been demonstrated elegantly in young infants, who seem capable of spontaneously attributing mental states to agents [86-88], and individuals with autism spectrum disorders who seem to have preserved explicit mental state reasoning, but impaired spontaneous mental state reasoning [89,90]. Though the relative consequences of implicit versus explicit ToM ability for social functioning are unknown, these data suggest these processes can be dissociated and studied separately. 

 Given these considerations, the goal of this study was to design a new ToM task – the *Short Story Task* (SST) - that improved upon the limitations of existing ToM measures. More specifically, we aimed to create a task that (a) was sensitive to individual differences in ToM ability and did not suffer from ceiling effects, (b) incorporated a range of mental states of differing complexity, including epistemic states, affective states, and intentions to be inferred from a first- and second-order level, (c) used ToM stimuli representative of real-world social interactions, (d) required participants to utilize social context when making mental state inferences, (e) exhibited adequate psychometric properties, and (f) was quick and easy to administer and score. 

 In considering appropriate stimuli for the task, literary fiction seemed like an ideal venue to test ToM ability. Fiction offers the opportunity to engage in simulated social experiences by transporting the reader into the social and mental life of story characters [91]. To make sense of story events and character actions, the reader is required to make inferences about the characters’ beliefs, emotions, desires, and intentions in the context of dynamically unfolding social scenarios. This idea is supported by several lines of research demonstrating that exposure to fiction is positively associated with greater ToM ability [92-95], the tendency to become emotionally transported into fictional stories is positively associated with an increase in empathy [96], and that the neural network recruited for ToM is largely overlapping with the network recruited during narrative comprehension [97]. 

Thus, in consultation with a Boston-based novelist, we used *The End of Something* [98], a short story by Ernest Hemingway, to test ToM ability. This story presents a nuanced interaction between a romantic couple (spoiler alert) that has a conflict and subsequently breaks up. As is typical of Hemingway’s fiction, the mental lives of the characters are not explicitly described, requiring readers to make a series of first- and second-order mental state inferences regarding epistemic states, affective states, and intentions, to understand story events and character actions. The prose is direct and easy to understand, reducing the potential impact of verbal ability on ToM reasoning. After reading the story, participants were asked a series of questions to gauge explicit mental state reasoning ability, spontaneous mental state inference, and, finally, comprehension of the non-mental story content to ensure adequate understanding of the prose. Performance on the mental state reasoning questions was evaluated with a scoring rubric completed by the experimenter. Points were assigned depending on the accuracy of the mental state inference and number of mental states taken into account. Spontaneous mental state reasoning was assessed with a single question that simply asked participants to summarize the story. The unprompted mention of mental states here theoretically reflects the salience of mental state information, and the propensity to think about mental states by the participants. 

 Towards the goal of assessing the concurrent validity of the SST as a measure of ToM ability, we employed two additional measures of social cognition: the Interpersonal Reactivity Index (IRI) [99,100] and the Eyes Task [38]. By testing for concurrent validity, we aimed to evaluate the extent to which SST performance is associated with these other well-established measures of social cognition, which were administered concurrently with the SST. We chose these particular measures for several reasons. First, both are ubiquitously employed in the social cognition and social neuroscience literature in studies of neurotypical and clinical populations. Second, both tasks have excellent psychometric properties [38,99,100], show concurrent validity with a range of other behavioral and neural measures of ToM [101-106], and distinguish clinical populations with established ToM deficits from non-clinical populations [1-3,38,107-109]. Furthermore, the Eyes Task is one of the few ToM tasks in which healthy adults show substantial variation in performance, and ceiling effects are not observed. Lastly, these two measures index different aspects of ToM than that tested by the SST. The IRI provides a self-reported measure of transportation into the mental and emotional lives of story characters, and an individual’s tendency to engage in different facets of perspective-taking and empathy in their own life. The Eyes Task provides an index of mental state *decoding* ability, which is the ability to identify mental states based on immediately available information (eyes in this case). This is different from the mental state *reasoning* demands of the SST which requires attributing mental states and then using that information to predict other mental states and actions [110]. Additionally, the Eyes Task requires analysis of visual images and thus tests ToM ability in a different sensory modality than the SST. Converging associations between the SST and these measures would provide strong support for the concurrent validity of the SST as a measure of ToM. We included the comprehension questions to provide further evidence regarding task validity. More specifically, the comprehension questions required similar verbal skills as the ToM questions, but did not test ToM ability. If the SST ToM scores are indexing some aspect of ToM ability, only these scores, and not the comprehension score, should be associated with the IRI and Eyes Task. 

 We tested for the following: (a) general psychometric properties of the SST including inter-rater reliability between independent judges scoring the mental state reasoning and spontaneous mental state inference question, and internal consistency, (b) relationships between mental state reasoning, spontaneous mental state inference, and comprehension of non-mental state information, (c) relationships between ToM ability as measured with the SST and demographic variables, as well as general intelligence, and, finally, (d) concurrent validity of the SST by examining the relationship between SST performance and scores on the IRI and Eyes Task.

## Materials and Methods

### Participants

 Seventy-four individuals (27 males, 47 females) were recruited from the greater Boston area via online advertisements and participated for monetary compensation. Participants ranged in age from 18 to 58 years (*M* = 27.8, *SD* = 9.6) and completed between 12 and 20 years of education (*M* = 15.7, *SD* = 1.9). As is typical of study samples in the Boston area, average IQ was quite high (*M* = 120.4, *SD* = 9.1) and ranged between 94 and 138 (IQ data were not collected for five participants who terminated their participation prior to the experiment being completed).

Inclusion criteria included being a native English speaker, IQ>70, and none of the following: neurological or major medical illness, lifetime Axis I/II DSM disorder, or current substance abuse problem. Of the 82 individuals who came to the lab to participate, six were excluded for meeting criteria for an Axis I DSM disorder and two were excluded for having a neurological abnormality. Lifetime psychopathology was assessed with the Mini-International Neuropsychiatric Interview (MINI) [111]. IQ was assessed using either the vocabulary and matrix reasoning subtests of the Wechsler Abbreviated Scale of Intelligence (WASI) [112] or the North American Adult Reading Test (NAART) [113]. Trained PhD students in clinical psychology administered these assessments. 

#### Ethics Statement

This study was approved by Harvard University’s Internal Review Board. All participants gave informed written consent before beginning the experiment.

### Short Story Task

#### Overview

In the Short Story Task (SST), participants read *The End of Something*, a short story by Ernest Hemingway [98], which presents a nuanced interaction between a romantic couple in which the male protagonist, Nick, starts an argument and breaks up with his girlfriend, Marjorie. Through the course of the story, the characters display sarcasm, non-verbal and indirect communication, higher-order emotions like guilt, and attempts to hide their intentions and feelings from one another. As is often the case in Hemingway’s fiction, the mental lives of the characters are not explicitly described. Thus, the reader is forced to make a series of first-order (i.e., inferring the belief or emotion of a single character) and second-order (i.e., inferring what one character thinks about another character’s belief, emotion, or action) mental state inferences in order to understand the ostensible mental lives of, and social interactions between the characters. Additionally, Hemingway’s prose is direct and easy to understand, reducing the potential impact of verbal ability on mental state reasoning. Hemingway, and this short story in particular, was chosen as the stimulus for this task for these aforementioned reasons with the consultation of a Boston-based novelist with a PhD in English and expertise in 20th Century American Literature (JPC). 

The Flesch Reading Ease Score (FRES) [114], which denotes the ease of reading comprehension on a 0-100 scale (higher scores indicate easier text), and the Flesch-Kincaid Grade Level (FKGL), which estimates the grade level at which text should be understood, indicated that *The End of Something* contained highly readable text (FRES = 92.7; for reference, the FRES of this manuscript’s abstract is 31.2) that should be understood by the average individual at a 3^rd^ grade reading level (FKGL = 2.8). The text is 1,427 words in length.

#### Administration

Before reading *The End of Something*, participants were given the following instructions:

“You are going to read a short story called *The* *End* *of* *Something*. The story is only a few pages, but take your time reading it. Try to get a sense of what happens and what the relationships are between the characters. After you’re finished, I’m going to ask you some questions and tape-record your responses. Do you have any questions before we begin?”

After reading the story, the experimenter asked a series of open-ended questions in a structured format. Participants were allowed to refer back to the story as needed, and were given a copy of the questions the experimenter asked in order to eliminate memory demands. First, the experimenter asked a set of questions regarding familiarity with the story to ensure that participants had no prior knowledge that might affect their responses. Four participants reported being familiar with the book that contained the short story – *In Our Time* – however, no participants reported having read *The End of Something* prior to the experiment. Participants were then given the following instructions:

“Now I’m going to ask you some questions about the story. Here is a copy of the questions I’ll be asking so you can read along. For most of the questions, there are no right or wrong answers and the questions can be answered with short responses. We’re also interested in the character’s thoughts, feelings and intentions when it applies to the question.”

We included this last sentence based on pilot data, which suggested that unless explicitly prompted, many participants were inclined to respond to questions by simply recounting the events of the story, instead of making inferences regarding what characters might be thinking or feeling.

An excerpt from *The End of Something* and an example mental state reasoning question follows: “He was afraid to look at Marjorie. Then he looked at her. She sat there with her back toward him. He looked at her back. ‘It isn’t fun any more. Not any of it.’” *Question*: Why is Nick afraid to look at Marjorie?

While administering the questions, the experimenter provided no feedback regarding the participant’s responses, and participants were free to respond at any length. Responses were recorded with a digital recorder and later transcribed by an undergraduate research assistant. The task was administered by either the first-author (DDF), another trained PhD student, or trained undergraduate research assistants. Administration of the task, including the time needed for the participant to read the story and the experimenter to administer the questions, typically took around 10 minutes. 

#### Questions and Scoring

Questions were designed to assess three factors: (a) five questions probed *comprehension* of the prose and story events (i.e., non-mental state content), (b) eight questions probed *explicit mental state reasoning* regarding story characters’ beliefs, emotions, intentions, and desires, and (c) one question assessed *spontaneous mental state inference* (Table 1). Scoring was completed by the first-author (DDF), using the transcriptions, according to a rubric. In order to evaluate inter-rater reliability, 25% of the transcripts were chosen at random and scored by a second independent rater (SHL).

**Table 1:** Description of Assessment Questions and Scoring Criteria in the Short Story Task.

	**Explicit Mental State Reasoning**	**Spontaneous Mental State Inference**	**Comprehension**
Number of Question(s)	8	1 (Participant is asked to summarize the story with no other prompt)	5
Individual Question(s) Scored	0, 1, 2	Yes, No	0, 1, 2
0	No MS inference; inaccurate MS reasoning	-	Patently inaccurate response
1	Consideration of only one (or few) perspectives, emotions, intentions; partial understanding of a character(s) MS	-	Partial understanding of non-mental story content
2	Consideration of several characters’ MS; second-order and higher MS inferences; accurate MS reasoning	-	Full understanding of non-mental story content
Yes/No	-	*Yes* = presence of unprompted MS inference regarding a story character’s beliefs, emotions, desires, or intentions; *No* = no presence of unprompted MS inference; response recounts only non-mental state story events	-
Total Score	0 - 16	-	0 - 10

Note. MS = Mental state.

For comprehension questions, the rubric was designed to assign more points depending on the accuracy of the participant’s response to questions probing the understanding of non-mental state story content. A *0* was assigned for responses that were patently inaccurate; *1* for responses that demonstrated partial understanding; and *2* for responses that demonstrated full understanding. *Comprehension scores*, which are the sum of scores from the five comprehension questions, can range from *0* – indicating no understanding of the story’s non-mental events and/or prose – to *10* – indicating excellent understanding of the story’s non-mental events and/or prose. This score was used to investigate whether mental state reasoning was associated with general understanding of the non-social aspects of the story. 

For explicit mental state reasoning questions (hereafter referred to as *mental state reasoning*), the rubric was designed to assign points based on the accuracy of the mental state inference, number of character perspectives/emotions taken into account (i.e., second-order inferences generally received more points than first-order inferences), and understanding of non-verbal/indirect communications (e.g., sarcasm and body language). Similar to the comprehension questions, each of these questions were assigned a value of *0*, *1*, or *2*, and an overall *mental state reasoning score* was calculated as the sum of points from the eight mental state reasoning questions. Thus, scores can range from *0* – indicating little to no understanding of the story characters’ mental states – to *16* – indicating excellent understanding of the story characters’ mental states. 

To assess *spontaneous mental state inference*, participants were asked a single question that simply asked them to summarize the story. Responses were coded for the presence or absence of a mental state inference. We had originally planned to code these responses not just for the presence versus absence of a mental state inference, but for the number of mental state inferences to use as a continuous variable. However, most participants provided very short summaries (1-3 sentences) and either made a single mental state inference (e.g., “Nick felt bad about breaking up with Marjorie.”) or none. Given that the summary question, which did not explicitly ask participants to make reference to the characters’ mental states, the unprompted mention of mental states should in theory reflect the relative importance and salience of mental states for the participant, and the propensity for the participant to think about mental states. This question was asked first, before the comprehension or mental state reasoning questions, in order not to prime participants with certain aspects of the story to summarize. We note however, that prior to asking this question, participants were told, as part of the instructions, “We’re also interested in the character’s thoughts, feelings and intentions when it applies to the question.” Thus, though the question itself does not specifically ask for the mention of mental states, the extent to which the mention of mental states here can be considered *truly* unprimed or spontaneous should be cautioned. 

Scoring each participant’s transcript took somewhere between 5 and 10 minutes depending on the length of the responses. All testing material, including the questions, scoring instructions, and rubric are provided in Text S1. 

### Interpersonal Reactivity Index

The Interpersonal Reactivity Index (IRI) is a 28-item self-report questionnaire that consists of the following four subscales: fantasy, perspective-taking, empathic concern, and personal distress [99,100]. The fantasy scale assesses the tendency to identify with fictional characters, become immersed in a narrative, and be mentally transported into a character’s mental and emotional life [92,93] (e.g., “When I am reading an interesting story or novel, I imagine how I would feel if the events in the story were happening to me.”). This subscale has been shown to be highly correlated with another measure of narrative immersion [93]. The perspective taking subscale assesses the tendency to adopt and reason about the mental states of others (e.g., “I sometimes try to understand my friends better by imagining how things look from their perspective.”). The empathic concern subscale assesses the tendency to consider the emotional states and experience sympathy for others (e.g., “I often have tender, concerned feelings for people less fortunate than me.”). The personal distress subscale assesses the tendency to experience negative affect in response to negative events experienced by others (e.g., “Being in a tense, emotional situation scares me.”). Each subscale consists of 7 items that are rated on a scale from 0 (*does not describe me well*) to 4 (*describes me very well*). 

### Reading the Mind in The Eyes Task

 In the Reading the Mind in The Eyes Task – Revised (Eyes Task) [38], participants view 36 pictures of the eye region of actors’ faces, and judge which of four adjectives best describes the mental state being expressed through the eyes. Photographs are centrally displayed on the computer screen and the four adjectives (one correct adjective and three distractors) are placed in the four corners of the screen. Participants respond with one of four buttons on a keyboard corresponding to each of the four adjectives. Participants were instructed to respond as accurately as possible. The 36 experimental trials are preceded by a single practice trial. Upon request, participants were provided with a list of the adjectives and their definitions used in the task. E-prime 2.0 was used to present the stimuli and collect accuracy data. 

### General Procedure

 Participants came to the lab to participate in one of several larger ongoing studies investigating social cognition in healthy and clinical populations. Upon entering the lab, all participants completed a general demographics questionnaire and the MINI to ensure eligibility. Most participants completed the IQ assessment and SST after these assessments and before the IRI and Eyes Task; however a portion of participants completed the IQ assessment, SST, IRI, Eyes Task, and other behavioral experiments/questionnaires unrelated to the current study, in a different order. One project did not collect IRI data, leaving 44 participants of the total sample with IRI data. After completing the experimental procedures, participants were debriefed and compensated for their time.

### Statistical Analysis

 Distributions of the comprehension score, mental state reasoning score, IRI, Eyes Task, and IQ were visually inspected for normality and outliers (±2.5 *SD* of the mean). Comprehension scores were substantially negatively skewed indicating a ceiling effect. Given this distribution, these data were dichotomized into two groups of individuals who attained a perfect score of 10 (*n* = 36) and those who scored below 10 (*n* = 38) for further analysis. We analyzed comprehension data in this way instead of performing a median split (*Mdn* = 9) as this would have resulted in substantially unequal group *n*’s 2 participants’ IQ scores were <2.5 *SD* of the mean and identified as outliers. These two values were Winsorized by replacing them with the next lowest non-outlying IQ score and subtracting 10% of that score to maintain variance. 

 Inter-rater reliability of the comprehension and mental state reasoning score was assessed with the intraclass correlation coefficient (ICC) using the 25% of transcripts scored by the independent judge. Inter-rater agreement on the presence/absence of a spontaneous mental state inference in the spontaneous mental state inference summary question was assessed with the kappa coefficient. Internal consistency of the comprehension and mental state reasoning questions was assessed with Cronbach’s alpha. We note that by emphasizing content validity in the questions asked, that is, by having participants reason about a range of different mental states from a first- and second-order level, alpha levels will be negatively impacted [115,116]. 

 Subsequent analysis addressed four main questions. First, we examined the relationship between the SST variables to examine whether individuals who made a spontaneous mental state inference were also better at explicit mental state reasoning, and whether spontaneous mental state inference and explicit mental state reasoning were related to understanding the non-mental aspects of the story (comprehension score). Second, we examined whether any of the SST scores were related to demographic variables, including age, gender, and education. Third, given the verbal demands of the task, we examined whether any of the ToM variables from the SST (mental state reasoning score, spontaneous mental state inference) were associated with general intelligence (IQ). Fourth, to assess concurrent validity of the SST, we investigated the relationship between SST scores, the IRI, and Eyes Task performance. For all of these analyses, the relationship between the mental state reasoning score and the other variables were evaluated with Pearson product-moment correlations, which are accompanied by 95% CIs (bias-corrected and accelerated) derived from 2,000 bootstrap samples. The relationship between the comprehension and spontaneous mental state inference score was evaluated between groups (i.e., those with/without a perfect comprehension score, and those who made/did not make a spontaneous mental state inference), with two-sample *t*-tests or chi-square tests where appropriate. Statistical significance was defined as *p* < .05, two-tailed for all analyses. Statistical analysis was performed with R (www.R-project.org). 

## Results

### Inter-Rater Reliability and Internal Consistency

 Inter-rater reliability was high for the mental state reasoning score (ICC = .98) as well as the comprehension score (ICC = .90). Inter-rater agreement on the presence versus absence of a spontaneous mental state inference was also high (kappa = .86). Unsurprisingly, given the range of content asked in the questions, internal consistency was low for the mental state reasoning questions (α = .54) and comprehension questions (α = .31). 

### SST

 For all SST scores, we visually inspected the distributions and conducted measures of skewness and kurtosis. For a unimodal normal distribution, a skew value of 0 indicates perfect symmetry of scores around the mean. Positive kurtosis values indicate that the distribution has relatively sharp peaks and fat tails relative to a normal distribution; negative kurtosis values indicate that the distribution has wide peaks and thin tails. 

**Figure 1:**
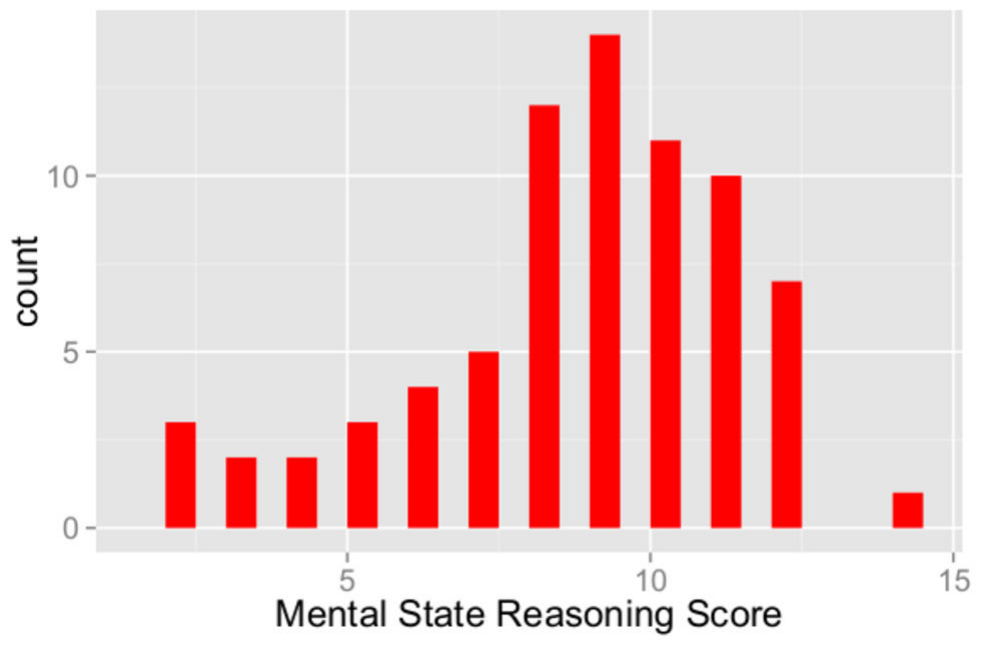
Distribution of the mental state reasoning score.

Mental state reasoning scores were relatively normally distributed with a slight negative skew (skew = -.72, kurtosis = .13) indicating an asymmetry in the distribution whereby the majority of scores were on the right side of the distribution (reflecting that the majority of individuals received scores of 8 out of 16 possible points or higher) (Figure 1). Importantly, there was substantial variation in performance across individuals with scores ranging from 2 to 14 (possible scores = 0-16), and no indication of a ceiling effect (0% of participants scoring 16/16 or 15/16). Mean score was 8.6 ± 2.6.

 Data from the spontaneous mental state inference summary question was collected from 70 participants (four participants were not asked the spontaneous mental state inference question due to experimenter error). 50% made at least one spontaneous mental state inference. Further analysis of this variable with other data proceeded with a dichotomized variable (i.e., individuals who did versus did not make a spontaneous mental state inference) as individuals tended to either make a single mental state inference or none. 

 Comprehension scores exhibited a substantial negative skew due to 48.6% of the participants performing at ceiling (skew = -.98, kurtosis = -.13). Performance ranged from 6 to 10 and the mean score was 9.0 ± 1.2 (possible scores = 0-10). Further analysis of the comprehension score was performed with the dichotomized variable; that is, those individuals who achieved a perfect score (*n* = 36) and those who did not (*n* = 38).

### Relationship Between the SST Variables

**Figure 2:**
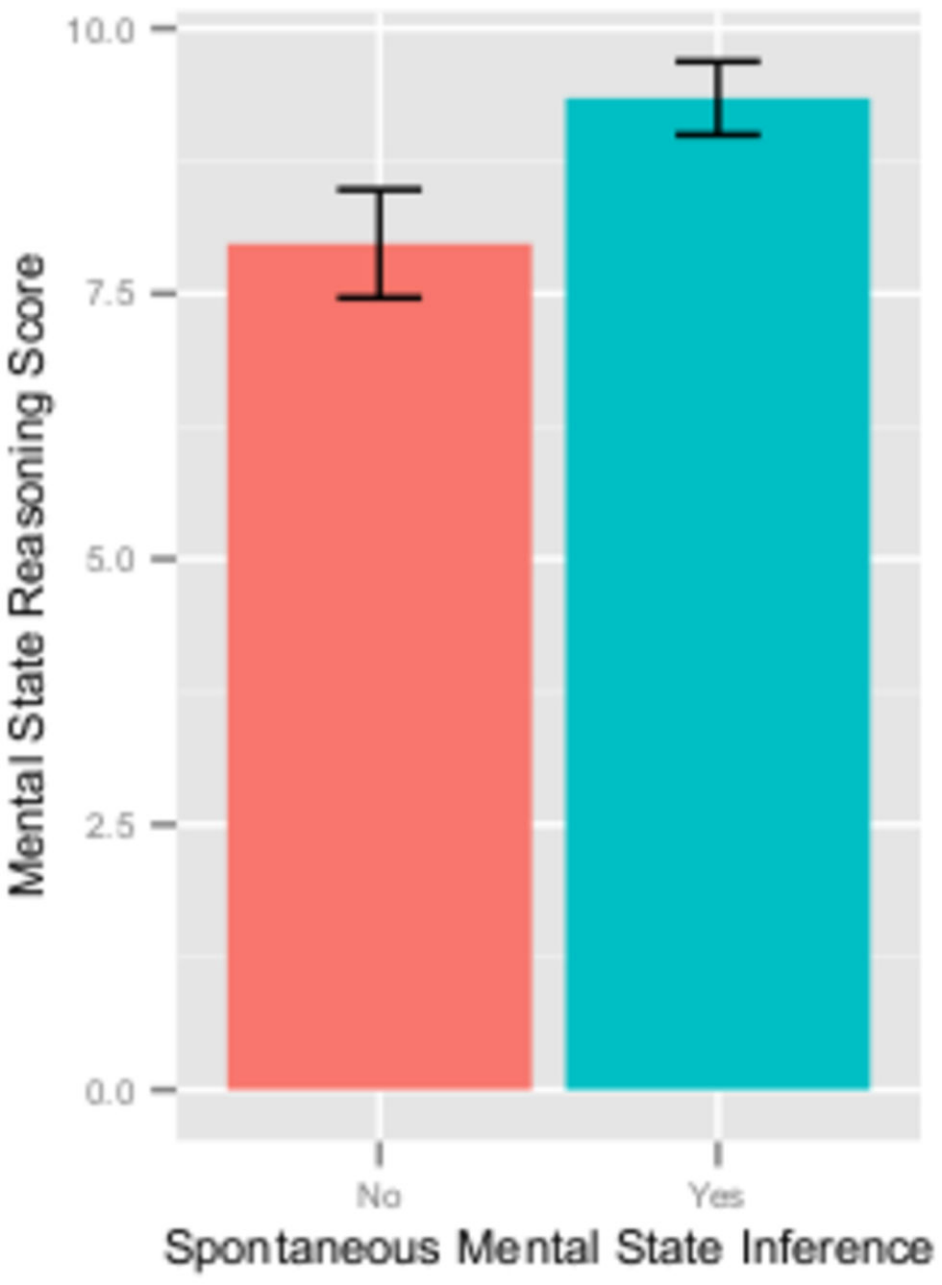
Mental state reasoning score as a function of spontaneous mental state inference. Mean mental state reasoning score of individuals who did (turquoise-colored bar) and individuals who did not (salmon-colored bar) make a spontaneous mental state inference in the summary question. Error bars depict standard error of the mean.

 Individuals who made a spontaneous mental state inference in the summary question had higher mental state reasoning scores (*M* = 9.3, *SD* = 2.0) compared to those individuals who did not make a spontaneous mental state inference (*M* = 8.0, *SD* = 3.0) (Figure 2). This difference was statistically significant, *t*(68) = 2.19, *p* = .032, Cohen’s d = .52. 

 Individuals who achieved a perfect score on the comprehension questions performed no differently on the mental state reasoning questions (*M* = 9.0, *SD* = 2.3) compared to those who had a score <10 (*M* = 8.1, *SD* = 2.9), *t*(72) = 1.47, *p* = .15, d = .34. Similarly, individuals who achieved a perfect score on the comprehension questions were equally as likely to make a spontaneous mental state inference (47.2%) as those who had a score <10 (47.4%), χ^2^(1, *N* = 74) = 0, *p* = 1.0.

### Relationship Between ToM Performance on the SST and Demographic Variables

 Mental state reasoning scores did not significantly differ by gender (*M*_males_ = 9.0, *SD* = 2.4; *M*_females_ = 8.3, *SD* = 2.8), *t*(72) = .97, *p* = .33, d = .24, nor did they correlate with age or education (Table 2). 

**Table 2:** Relationship Between Mental State Reasoning Score, Demographic Variables, IQ, and Social Variables.

**Variable**	***r***	***p***	**95% CI**
Age	-.12	.29	[-.36, .07]
Education	.19	.11	[-.06, .41]
IQ	**.24**	**.047**	**[.02, .50]**
IRI-Fantasy	**.37**	**.012**	**[.17, .53]**
IRI-Perspective Taking	-.07	.65	[-.35, .23]
IRI-Empathic Concern	-.07	.67	[-.29, .14]
IRI-Personal Distress	.05	.76	[-.36, .35]
Eyes Task	**.49**	**< .0001**	**[.27, .68]**

Note. Bold values denote statistical significance at *p* < .05.

 The number of males who made a spontaneous mental state inference (61.5%) did not significantly differ from the number of females who made a spontaneous mental state inference (43.2%), χ^2^(1, *N* = 70) = 2.20, *p* = .14. Similarly, neither age nor education differed between those who made a spontaneous mental state inference and those who did not (Table 3).

**Table 3:** Relationship Between Spontaneous Mental State Inference, Demographic Variables, IQ, and Social Variables.

**Variable**	**Spontaneous Mental State Inference Group**	**No Spontaneous Mental State Inference Group**	**Between-Group Difference**
Age (years)	26.4 (8.3)	29.1 (11.2)	*t*(68) = 1.15, *p* = .25, d = .27
Education (years)	15.6 (1.8)	15.8 (2.0)	*t*(68) = .44, *p* = .66, d = .11
IQ	121.8 (8.3)	119.5 (9.2)	*t*(63) = 1.07, *p* = .29, d = .26
IRI-Fantasy	17.1 (5.4)	15.9 (5.5)	*t*(42) = .75, *p* = .46, d = .23
IRI-Perspective Taking	20.3 (5.2)	18.5 (4.6)	*t*(42) = 1.23, *p* = .23, d = .37
IRI-Empathic Concern	21.2 (5.3)	20.5 (4.1)	*t*(42) = .51, *p* = .61, d = .15
IRI-Personal Distress	9.9 (5.1)	10 (5.5)	*t*(42) = .06, *p* = .95, d = .02
Eyes Task (% correct)	78.4 (7.3)	76.3 (11.6)	*t*(62) = .85, *p* = .40, d = .21

Note. Values represent means and standard deviations in parentheses. All tests were performed between individuals who did and those who did not make a spontaneous mental state inference.

### Relationship Between ToM Performance on the SST and IQ

**Figure 3:**
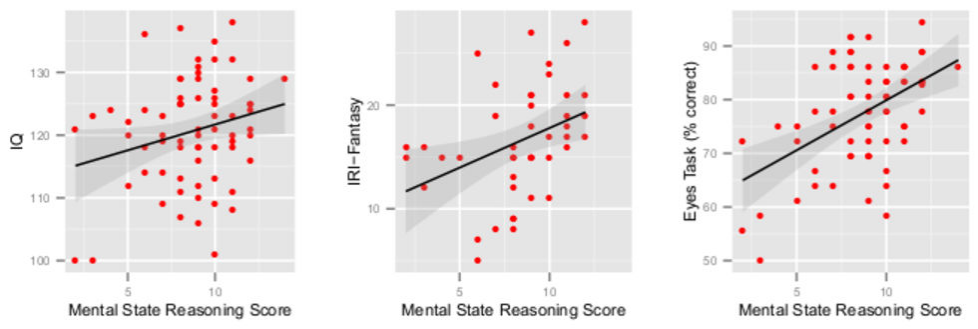
Relationship between mental state reasoning scores and IQ, fantasy, and eyes task scores. Shaded area represents 95% CIs.

 Mental state reasoning scores exhibited a statistically significant relationship with IQ such that higher mental state reasoning scores were associated with higher IQ (Table 2, Figure 3). Sixty-five participants had IQ data and were asked the spontaneous mental state inference question. There was no difference in IQ between individuals who made a spontaneous mental state inference and individuals who did not (Table 3). 

### Concurrent Validity of the SST and Other Measures of Social Cognition

 In order to evaluate concurrent validity of the SST, we examined ToM performance on the SST with the IRI and Eyes Task. IRI data were collected for 44 participants. Performance on the Eyes Task ranged from 50 to 94.4% correct. Mean performance was 77.4 ± 9.8% correct, which is similar to other studies of non-clinical populations (e.g., [38,103,117]). 

Mental state reasoning scores on the SST exhibited a statistically significant relationship with the fantasy subscale such that better performance was associated with higher fantasy scores (Table 2, Figure 3). This relationship was not found with the other IRI subscales. Mental state reasoning scores also exhibited a statistically significant relationship with the Eyes Task such that better performance was associated with greater accuracy on the Eyes Task (Table 2, Figure 3). This relationship was preserved in the subset of 43 participants who had IRI and Eyes Task data, *r*(41) = .59, *p* < .0001, 95% CI [.32, .77]. 

Performance on the Eyes Task was significantly correlated with IQ, *r*(66) = .24, *p* = .048, 95% CI [-.03, .50]. Thus, in order to evaluate the relative contribution of IQ to the relationship between SST mental state reasoning and Eyes Task performance, we conducted a partial correlation controlling for IQ, which did not alter the relationship, *r*(65) = .45, *p* < .0001, 95% CI [.26, .62]. Fantasy scores were not associated with IQ, *r*(42) = -.13, *p* = .39, 95% CI [-.41, .15]. Controlling for IQ also did not alter the relationship between mental state reasoning and IRI fantasy scores, *r*(41) = .42, *p* = .003, 95% CI [.18, .63].

To further evaluate whether mental state reasoning, specifically, was associated with the fantasy scale and performance on the Eyes Task, as opposed to some other aspect of the task such as general reading or verbal ability, we looked at these measures as a function of comprehension score. Fantasy scores in the perfect comprehension group (*M* = 16.6, *SD* = 4.7) did not differ from those in the <10 group (*M* = 16.5, *SD* = 6.0), *t*(42) = .06, *p* = .95, d = .02. Similarly, Eyes Task performance in the perfect comprehension group (*M* = 78.1, *SD* = 9.5) did not differ from those in the <10 group (*M* = 76.7, *SD* = 10.1), *t*(66) = .59, *p* = .56, d = .14.

Lastly, we evaluated whether making a spontaneous mental state inference on the summary question was also associated with the IRI and Eyes Task. Individuals who made a spontaneous mental state inference on the summary question had higher scores on all subscales of the IRI (particularly perspective-taking, d = .37) except personal distress, and the Eyes Task; however, none of these differences were statistically significant (Table 3). 

## Discussion

 Here, we report findings from the Short Story Task (SST), a new measure of ToM ability for adults. This task was designed to improve upon limitations inherent in existing ToM tasks. More specifically, the SST was designed to provide a relatively sensitive metric of ToM ability in adults, capable of picking up on individual differences and normal variation in ToM ability, with assessment procedures that were quick and easy to administer and score reliably. Furthermore, the task stimulus (the short story) was representative of a real-world, dynamically unfolding, complicated social scenario that required the application of social knowledge, and participants answered questions that assessed both explicit mental state reasoning and spontaneous mental state inference. 

We found that on our measure of explicit mental state reasoning, participants demonstrated substantial variation in performance across almost the full range of possible scores. There was no indication of a ceiling effect as no participant received a perfect score of 16 out of 16 possible points. This variation suggests that the SST is sensitive to individual differences in ToM ability; a clear improvement from many of the existing ToM tasks. The improvement in sensitivity could be related to the fact that participants were asked to reason about a dynamically unfolding social scenario that required the consideration of the social context. This scenario was far more complicated in terms of the social context, emotions, and intentions ostensibly experienced by the story characters compared to the simple vignettes used in other ToM tasks. Furthermore, the scoring rubric was tailored to award higher scores for responses that were not only more accurate, but considered the mental life of several characters at once. 

On the spontaneous mental state inference question, half of the participants made an unprompted mention of a character’s belief, emotion, desire, or intention. Interestingly, participants who made a spontaneous mental state inference performed better on the explicit mental state reasoning questions, suggesting that the increased salience and propensity to think about mental state information is associated with better conscious reasoning about mental states. Though data from young infants [86-88] and individuals with autism spectrum disorders [89,90] suggest that the capacity to spontaneous attribute mental states may be relatively independent of explicit mental state reasoning, our data suggest that, at least in healthy adults, these two processes may be related. Furthermore, the fact that performance is related on these two SST measures, which theoretically index aspects of ToM ability, provides additional evidence of the SST as measuring the underlying construct of ToM. We also note that a higher percentage of males (61.5%) made a spontaneous mental state inference than females (43.2%). Though not statistically significant (*p* = .14), this pattern of results is not typical with tasks assessing aspects of ToM and empathy [32,38,118] perhaps, in part, because of the shared variance between ability on these measures and autistic/schizotypal traits, which may be higher in males [119-121]. With that said, many of these findings are with tasks testing explicit mental state reasoning; less is known about gender differences in spontaneous mental state reasoning.

We examined several additional psychometric properties of the SST, including inter-rater reliability, concurrent validity, and internal consistency. Inter-rater reliability was excellent for the mental state reasoning and comprehension scores, as well as judgments on the presence versus absence of a spontaneous mental state inference. This highlights the SST as a measure that is relatively easy to score reliably. We tested whether the SST scores exhibited concurrent validity with other commonly used measures of social cognition that exhibit adequate psychometric properties. We found that greater performance on the mental state reasoning questions was positively associated with scores on the fantasy scale of the IRI and performance on the Eyes Task. The fact that the IRI and Eyes Task differs from the SST on several important dimensions (i.e., the IRI being self-report and the fantasy scale measuring the tendency to become immersed in the mental life of fictional characters; the Eyes Task testing mental state decoding) provides strong support for the validity of the SST as measuring the underlying construct of ToM ability. Internal consistency was low for the mental state reasoning questions, which is not surprising given the several different facets of ToM ability probed by the questions (e.g., inferences regarding epistemic, affective, intentional states, first- and second-order inferences, etc.). Here, adequate content validity might have made some questions more difficult than others, decreasing this statistic [115,116], which in our opinion is a worthwhile tradeoff. Furthermore, the alpha values observed for the mental state reasoning score are similar or superior to those derived from other ToM ability tests (e.g., [122,123]). 

The correlation between the fantasy scale and SST mental state reasoning performance is consistent with other studies showing significant positive inter-relationships among the fantasy scale, Eyes Task performance, and exposure to fiction. More specifically, healthy adults who report greater exposure to fiction report higher scores on the fantasy scale (but not other subscales of the IRI) and perform better on the Eyes Task, even after controlling for demographic and personality variables [92,93]. Additional research has demonstrated that greater transportation into the emotional life of fictional characters is associated with increased empathy over time [96]. The current data provide additional evidence that individuals who become immersed in the mental life of fictional characters perform better on ToM tasks. This raises the intriguing possibility that fiction reading actually improves ToM ability. Though our data cannot speak to causation, findings from preschoolers demonstrate that increased exposure to storybooks predicts better ToM ability [94]. Given that preschool children are unable to control their access to the type of media they are exposed to, self-selection effects (i.e., individuals who are better at ToM simply enjoy reading fiction more) are unlikely. Furthermore, its been shown that adults randomly assigned to read a short piece of literary fiction outperform individuals assigned to read non-fiction on a variety of ToM tasks, including the Eyes Task [124]. The way in which fiction reading could improve ToM may occur through several routes. One possibility is that fiction provides an opportunity to simulate the character’s social experience and thus provide a forum for the reader to practice reasoning about others’ mental states, and using that information to imaginatively implement appropriate social behaviors. Another possibility is that fiction helps readers build their social knowledge by exposing them to social rules and contingencies presented in the context of the story [91,92]. If reading fiction does indeed improve ToM ability, it would have obvious clinical applications, as it could be an easily implemented and cost-effective intervention for individuals with ToM impairment. Additional research has demonstrated that brief exposure to short fictional stories decreases one’s need for cognitive closure, specifically the need for order and structure and discomfort with ambiguity [125]. Such decreased rigidity regarding intolerance of uncertainty may be a similar skill to that trained by many interventions that aim to improve impaired social cognitive abilities, such as Cognitive Enhancement Therapy [126], Social Cognition and Interaction Training [8], and Social Cognitive Skills Training [127]. These interventions aim, in part, to reduce “jumping to conclusions” (i.e., forming rigid interpretations not amenable to disconfirming evidence) regarding what other individuals may be thinking, feeling, or intending, and foster an individual’s ability to flexibly evaluate multiple interpretations of other individuals’ behavior. Thus, in addition to potentially improving ToM ability per se, fiction reading may additionally cultivate more general skills that subserve social cognitive ability.

Mental state reasoning scores and spontaneously inferring a character’s mental state were unrelated to understanding the non-mental aspects of the story, suggesting that our questions isolated ToM ability and not general reading ability. With that said, despite our efforts to reduce the non-social cognitive demands of the task (i.e., verbal ability, memory) by using a story with relatively easy-to-read prose, allowing participants to refer back to the story as needed, and providing them with the questions, mental state reasoning scores exhibited a significant, although weak, positive association with IQ. We found a similar positive relationship between IQ and our other social-cognitive ability measure, the Eyes Task. Positive associations between IQ or verbal ability and ToM have been found in studies with children [128,129], individuals with schizophrenia [1,34], and individuals with autism spectrum disorders [130-134]. This relationship becomes especially apparent when ToM is tested with verbal stimuli. Similar to our study, given the verbal demands of the SST, it is not surprising that there exists some relationship between IQ and ToM ability as measured here. Importantly, despite this relationship, we found SST task performance to be related to the Eyes Task and the fantasy scale even after controlling for IQ. Additionally, the fact that comprehension scores were not related to either the IRI or Eyes Task provides further support that the mental state reasoning score is indexing ToM ability and not some peripheral cognitive process or ability that is concomitant with mental state reasoning.

 Several limitations are notable. First, our measure of spontaneous mental state inference, while associated with performance on the mental state reasoning questions, was not associated with performance on either the IRI or Eyes Task. It is noteworthy that individuals who did make a spontaneous mental state inference had higher scores on several IRI subscales and the Eyes Task of reasonable effect sizes (e.g., perspective-taking d = .37); however, statistical significance (*p* < .05) was not achieved. We probed spontaneous mental state inference with a single question and coded responses into a dichotomous variable, all of which may have limited the sensitivity of the measure and our ability to pick up on individual differences. Spontaneous mental state inference may be better evaluated with tasks that capture a wider range of performance. Eye-tracking patterns during visual inspection of social images, for example, may be a better proxy of real-world social interaction in which mental state information is often initially processed through gaze following [89]. It will also be important to tease apart the spontaneous mention of mental states *relative* to the spontaneous mention of non-mental state content (e.g., [135]); something which we were unable to investigate here due to the limited mention of mental states and short overall responses. Furthermore, as part of the instructions, which were administered prior to this question, participants were asked to consider the story characters’ thoughts, feelings, and intentions when it applied to the question. As a consequence, it is unclear whether the mention of mental states here can be considered truly spontaneous. With that said, only half of participants made a mental state inference to this question suggesting that the mention of mental states here was not considered mandatory (as could have been interpreted from the instructions), and reflects differences in the salience or importance of mental states to the participant as central to the story’s events. Second, we do not have data speaking to the predictive validity of the SST, specifically concerning real-world social outcomes. Given the relationship between ToM and social functioning, we would expect SST ToM scores to predict social skills and social success both longitudinally and cross-sectionally. Experience sampling methods that allow for repeated, momentary assessment of real-world social interaction would be well suited to address this important question. Lastly, we tested the SST with a relatively small number of participants. As a consequence, many of the analyses may have been underpowered (e.g., the correlations between SST scores and IRI scores where *n* = 44 or less) and should be interpreted with caution. 

 In summary, the SST represents a new task for assessing ToM ability in adults that is sensitive to individual differences, correlates with other well established measures of ToM ability, and is relatively quick and easy to administer and score. Given the diversity of contexts in which mental state attributions are made [25], we recommend the use of this task with other measures of social cognition that test ToM in these different contexts. There is still much progress to be made in the assessment of ToM and we hope that the use of this task will be fruitful in that endeavor. 

## Supporting Information

Text S1 **Short Story Task Administration and Scoring Materials.**
(DOCX)
