# Supplementary material for: Using Fiction to Assess Mental State Understanding: A New Task for Assessing Theory of Mind in Adults
Source: PLoS One. 2013 Nov 7;8(11):e81279. doi: 10.1371/journal.pone.0081279 (PMC3820595; doi:10.1371/journal.pone.0081279)
Supplement: Text S1 — Short Story Task Administration and Scoring Materials. (DOCX) [file pone.0081279.s001.docx]

**Text S1. Short Story Task Administration and Scoring Materials.**

**Instructions to Participant:**

Now you are going to read a short story called *The End of Something.* The story is only a few pages, but take your time reading it. Try to get a sense of what happens and what the relationships are between the characters. After you’re finished, I’m going to ask you some questions and tape-record your responses. Do you have any questions before we begin?

**After story is read:**

Have you read this story before? [yes | no]

IF YES

How long ago did you read it?

How well do you remember the story?

Did you read it for school or pleasure?

IF SCHOOL

What grade were you in?

What class was it for?

Is the story familiar to you? [yes | no]

IF YES

Do you know anything about the story? What do you know about it?

Have you discussed the story with anyone?

**Instructions to Participant:**

Now I’m going to ask you some questions about the story. Here is a copy of the questions I’ll be asking so you can read along. For most of the questions, there are no right or wrong answers and the questions can be answered with short responses. We’re also interested in the character’s thoughts, feelings and intentions when it applies to the question^[[1]](#footnote-1)^.

- Turn on tape recorder and put close to participant.
- Say *subject ID* and *short story task* into recorder.
- DO NOT give feedback and DO NOT score as you go.

**Questions**

1. In just a few sentences, how would you summarize the story?
2. What do Nick and Marjorie observe on the shoreline as they are rowing to the point to set their fishing lines?
3. What does Nick mean when he says, “They aren’t striking?”
4. Nick and Marjorie have a pail of perch for what purpose?
5. Do Marjorie’s actions suggest that she is experienced or inexperienced at fishing? What makes you say that?
6. Why does Nick say to Marjorie, “You know everything”?
7. Why does Marjorie reply, “Oh Nick, please cut it out! Please, please don’t be that way!”?
8. Why is Nick afraid to look at Marjorie?
9. What does Nick mean when he says, “It isn’t fun anymore”?
10. Why does Marjorie sit with her back toward Nick when she asks, “Isn’t love any fun?”?
11. Why does Marjorie take the boat and leave and what is she feeling at that moment?
12. Who is Bill and what does he reveal when he asks Nick, “Did she go alright? … Have a scene?”?
13. What is Nick feeling when he says, “Oh, go away, Bill! Go away for a while”?
14. The story is called “The End of Something.” What is the title referring to?

**Scoring Instructions and Rubric**

**General Instructions:**

- If the participant provides a response that is patently wrong, i.e., a “spoiled” response, unless they take it back or qualify their response in a way that would give them a score of 1 or 2 (i.e., back-track on their spoiled response), it should be given a score of 0.
- If the participant provides a response that would be scored a 1 and a 2, score it as a 2.
- If the participant provides a response that is accurate, but does not answer the question, it should be scored a 0.

**1) *SPONTANEOUS MENTAL STATE INFERENCE*: In just a few sentences, how would you summarize the story?**

The free-response question is coded for the presence or absence of a mental state inference. If the participant does make a mental state inference (i.e., about a belief, thought, desire, intention, goal, emotion), then this is coded as “1.” If the participant does not make a mental state inference, then this is coded as “0.”

**Example mental state inferences:**

*Nick felt bad* about breaking up with Marjorie.

*Nick intended* to break up with Marjorie all along.

*Marjorie knew* Nick was upset about something.

*Nick no longer loved* Marjorie.

*Nick chooses/decides* to end the relationship

- Responses regarding the participant’s own mental states, e.g., “*I think… I knew…*” are NOT counted as a mental state inference. The inference must be about a character in the story.
- Responses regarding the overall tone of the story, e.g., “It’s a *sad* breakup story,” which are not about a character’s mental state, DO NOT count as a mental state inference.
- Responses that speak to the romantic relationship, e.g., “The story is about a couple in a romantic relationship… end their romantic relationship… it was about a guy and a girl breaking up…Nick breaks up with her,” that doesn’t clearly get at a character’s mental state, DO NOT count as a mental state inference.

**2) *COMPREHENSION*: What do Nick and Marjorie observe on the shoreline as they are rowing to the point to set their fishing lines?**

**2** – An abandoned (lumber) mill; the white limestone foundations of the (lumber) mill; a broken down/old (lumber) mill; an abandoned (lumbering) town; Horton’s Bay

**1** – Description of shoreline; swampy growth/swampy meadow; (lumber) mill; AND no mention of town or buildings/structures

**0** – None of the responses in 2 or 1

**3) *COMPREHENSION*: What does Nick mean when he says, “They aren’t striking?”**

**2** – The fish aren’t eating the bait; the fish aren’t feeding; fishing isn’t going well

**1** – Any response that mentions fishing, but does not speak to the fact that the fish aren’t taking the bait

**0** – Any response that doesn’t convey that the fish aren’t taking the bait or that fishing is not going well or that doesn’t mention fishing at all

**4) *COMPREHENSION*: Nick and Marjorie have a pail of perch for what purpose?**

**2** – Bait; catching fish

**1** – Any response that conveys understanding of its use for some aspect of fishing without being explicit about its function as bait for catching fish

**0** – Any response that doesn’t convey understanding of its use for fishing

**5) *COMPREHENSION*: Do Marjorie’s actions suggest that she is experienced or inexperienced at fishing? What makes you say that?** *Make sure subject attempts to justify their answer

**2** – Experienced/somewhat experienced/somewhat inexperienced (depending on whether they interpret her skills relative to Nick versus most people) with following possible justifications: she says she loves fishing, can prepare the bait, holds the line in her mouth, knows how to steer the boat, knows where to cast the line; she can prepare the bait, although perfectly or as good as Nick, often asks Nick if she’s doing things correctly and Nick often corrects her; only somewhat experienced because she needs to constantly ask Nick and doesn’t do things as well as Nick

**1** – Somewhat experienced/inexperienced, but with poor justification (i.e., none of the justifications mentioned above); inexperienced with justifications mentioned above

**0** – Inexperienced without qualifying response; if response is “inexperienced,” but then back tracks after giving justification and changes to somewhat experienced/inexperienced, should be counted as **1** or **2** depending on justification

**6) *EXPLICIT MENTAL STATE REASONING:* Why does Nick say to Marjorie, “You know everything”?**

**2** – He’s being sarcastic/cynical/intentionally mean AND wants to get Marjorie

upset/sad/mad/annoyed; provoke a fight or provoke Marjorie so that she breaks up with him so he can blame the breakup on her

**1** – He’s unhappy with the relationship; wants to end the relationship; He’s annoyed/nervous about the situation/impending breakup; he’s being sarcastic/cynical (no mention of consequences, i.e. what Marjorie’s reaction will be)

**0** – He thinks Marjorie is a know-it-all; He’s just being mean; He’s a mean person

**7) *EXPLICIT MENTAL STATE REASONING:* Why does Marjorie reply, “Oh Nick, please cut it out! Please, please don’t be that way!”?**

**2** – She knows Nick is trying to provoke a fight/intentionally giving her a hard time and doesn’t want to have a confrontation; she senses that Nick might break-up with her

**1** – They have had this type of conversation before; she doesn’t want to fight; she doesn’t want to ruin a nice day

**0** – None of the responses in 2 or 1

**8) *EXPLICIT MENTAL STATE REASONING:* Why is Nick afraid to look at Marjorie?**

**2** – *response needs to reference Marjorie’s possible reaction to what he’s saying;* He knows she

is hurt/upset by his comment, and he is afraid of her reaction/doesn’t want to see the hurt in

her face; is afraid of her judgment of him

**1** – *some response that conveys his feelings without referencing how Marjorie’s reactions affect his feelings;* he is uncomfortable with the way the conversation is heading; he feels guilty/shameful/sad; he’s about to break up with her and it’s easier not to look at her; he’s afraid he’s making the wrong decision by breaking up with her

**0** – he doesn’t want Marjorie to see *his* reaction; none of the responses in 2 or 1

**9) *EXPLICIT MENTAL STATE REASONING:* What does Nick mean when he says, “It isn’t fun anymore”?**

**2 –** He’s tired of the relationship; he wants to end the relationship; their relationship/love isn’t fun anymore; the relationship is no longer enjoyable/bringing him happiness (response can mention fishing as example of how nothing they do together is fun any longer; response can also be “Could be the relationship, or could be fishing”); being with her (as long as “being with” refers to being with her more globally, like in the context of the relationship, and not on this specific fishing trip or being with her in this very moment)

**1 –** A response that only partially captures the understanding that he’s dissatisfied with

their relationship or alludes to the relationship without explicitly acknowledging dissatisfaction with the relationship per se, e.g. being around her/spending time with her

**0** – going fishing with her ONLY; None of the responses in 2 or 1

**10) *EXPLICIT MENTAL STATE REASONING:* Why does Marjorie sit with her back toward Nick when she asks, “Isn’t love any fun?”**

**2 –** She knows Nick is about to end their relationship; she is afraid of his answer because she knows it’s going to be bad/hurtful/not what she wants to hear; she’s trying to protect herself from his response because she knows it’s bad; she’s afraid of showing him how vulnerable/hurt/upset she is

**1 –** She’s upset/mad/afraid of crying; she is uncomfortable with the conversation

**0** – None of the responses in 2 or 1

**11) *EXPLICIT MENTAL STATE REASONING:* Why does Marjorie take the boat and leave (*1 point*) and what is she feeling at that moment (*1 point*)?** *Make sure subject attempts to answer both questions

**2 –** She realizes her relationship with Nick is over, she wants space to herself, she doesn’t want Nick to see her upset/vulnerable AND she’s feeling upset/sad/angry/disappointed/rejected (negative affect)

**1 –** Any response that doesn’t fully and accurately convey Marjorie’s understanding that the relationship is over AND feels anger/disappointment/sadness etc. (negative affect) – i.e., the answer needs to convey understanding of Marjorie’s negative affect, but does not convey Marjorie’s understanding that the relationship is over or vice-versa

**0 –** None of the responses in 2 or 1

**12) *EXPLICIT MENTAL STATE REASONING:* Who is Bill and what does he reveal when he asks Nick, “Did she go alright? … Have a scene?”?**

**2 –** Bill is a friend/lover of Nick; Bill knew that Nick was going to break up with Marjorie and would likely be upset/angry/fight with Nick in response

**1 –** Any response that misidentifies Bill’s relation to Nick (or just says Bill has some form of relationship with Nick/Bill knows Nick) OR doesn’t acknowledge that Bill knew something in advance

**0 –** None of the responses in 2 or 1

**13) *EXPLICIT MENTAL STATE REASONING:* What is Nick feeling when he says, “Oh, go away, Bill! Go away for a while”?**

**2 –** He feels guilty/sad/upset about hurting Marjorie (negative affect in relation to the fact that he just broke up with Marjorie) and needs his space to process things/doesn’t want to talk about it with Bill

**1 –** Any response that describes that Nick is experiencing negative affect, but doesn’t put the affect in the context of him breaking up with/hurting/upsetting Marjorie (answer can include he wants to be left alone), e.g. he’s upset/sad/angry and wants to be left alone

**0 –** ONLY he wants to be left alone/he wants some space to think about things/process things; none of the responses in 2 or 1

**14) *COMPREHENSION*: The story is called “The End of Something.” What is the title referring to?**

**2** – the end of Nick and Marjorie’s relationship; the end of innocence or happiness; the end of being able to blame someone else for your own actions/decisions; the end of one of these things and (but doesn’t have to include) the end of Horton’s Bay as a bustling lumbering town/the end of the mill

**1 –** mentions ONLY the end of something related to Horton’s Bay/the mill

**0** – None of the responses in 2 or 1

1. For future investigations, we would suggest changing this instruction to, “We’re also interested in what you believe to be the character’s thoughts, feelings, and intentions when it applies to the question.” We also recommend giving these instructions *after* question 1 (below) is asked. [↑](#footnote-ref-1)
